# Complete Genome Sequence of a Rabies Virus Isolate from a Ferret Badger (*Melogale moschata*) in Jiangxi, China

**DOI:** 10.1128/genomeA.00192-13

**Published:** 2013-06-13

**Authors:** Jinghui Zhao, Shoufeng Zhang, Ye Liu, Fei Zhang, Rongliang Hu

**Affiliations:** Laboratory of Epidemiology, Institute of Military Veterinary, Academy of Military Medical Sciences, Key Laboratory of Jilin Province for Zoonosis Prevention and Control, Changchun, Jilin, China

## Abstract

The genome of ferret badger rabies virus JX09-17(fb), isolated in Jiangxi Province, China, in 2009 has been completely sequenced. The genomic length is 11, 923 nucleotides (nt) with an overall organization similar to that of other rabies virus isolates. JX09-17(fb) is closely related to Chinese epidemic canine isolates in clade I.

## GENOME ANNOUNCEMENT

Rabies virus is the prototypical neurotropic virus and has a small, negative-stranded RNA genome of about 12 kb, which encodes five proteins in the order 3′-N–P-M-G-L-5′ (1). Rabies remains a public health problem worldwide because of its high fatality rate, global distribution, and human health costs. In China, ferret badgers (*Melogale moschata*) are a major reservoir. Our previous epidemiological study showed that ferret badger (FB) rabies has likely developed as an independent enzootic cycle of rabies infestation in southeast China (2, 3).

JX09-17(fb) was isolated from the brain of a ferret badger captured in Fuzhou, Jiangxi Province, China, in February 2009 (4). Initial phylogenetic analysis of full N and G gene sequences confirmed that this virus was most closely related to the other epidemic canine isolates from China and placed in clade I (4, 5). Of note is that JX09-17(fb) contains an R_333_Q substitution within its glycoprotein antigenic site III while still possessing high virulence in suckling and adult mice (4). The amino acids R_333_ and K_333_ on the glycoprotein of the rabies virus (RV) have been identified as necessary for virulence in adult mice (6, 7); however, several recent reports showed that RV pathogenicity is not absolutely dependent on substitution at amino acid position 333 of the glycoprotein (8–12).

In the present report we provide the complete genomic sequence of JX09-17(fb). Total RNA of infected mouse brains was extracted with TRIzol (Invitrogen, Carlsbad, CA) according to the manufacturer’s instructions. Reverse transcription (RT)-PCR was performed with nine pairs of primer sets designed based on rabies strain BD06 sequences available from GenBank. The PCR products were purified and cloned into the pMD18-T vector (TaKaRa, Dalian, China). Selected positively identified clones were sequenced at least twice in both directions (Nanjing Genscript Biological Technology Co., Ltd., China). Similarity scores and percentage identities were determined using DNASTAR.

The whole-genome length of JX09-17(fb) was found to be 11,923 nucleotides, with genomic organization similar to previously sequenced rabies virus genomes: N gene, 1, 353 nt; P gene, 894 nt; M gene, 609 nt; G gene, 1, 575 nt; L gene, 6,387 nt. Compared with Chinese street isolates and vaccine strains, the strain JX09-17(fb) showed 83 to 97% nucleotide identity. JX09-17(fb) was closely related to some Chinese epidemic isolates (BD06, ZJ-QZ, GC07, and Shaanxi-HZ-6) from dogs and genetically independent of other ferret badger isolates (JX08-45, JX08-47, JX08-48, ZJ-LA, and F04) in Jiangxi and Zhejiang province.

### Nucleotide sequence accession number.

The complete genomic sequence of JX09-17(fb) has been deposited in GenBank under the accession number KC762941.

## References

[1] Schnell MJ McGettigan JP Wirblich C Papaneri A 2010 The cell biology of rabies virus: using stealth to reach the brain. Nat. Rev. Microbiol. 8:51–61 19946287 10.1038/nrmicro2260

[2] Zhang S Tang Q Wu X Liu Y Zhang F Rupprecht CE Hu R 2009 Rabies in ferret badgers, southeastern China. Emerg. Infect. Dis. 15:946–949 19523299 10.3201/eid1506.081485 PMC2727325

[3] Liu Y Zhang S Wu X Zhao J Hou Y Zhang F Velasco-Villa A Rupprecht CE Hu R 2010 Ferret badger rabies origin and its revisited importance as potential source of rabies transmission in Southeast China. BMC Infect. Dis. 10:234 20691095 10.1186/1471-2334-10-234 PMC2927599

[4] Zhang S Zhao J Liu Y Fooks AR Zhang F Hu R 2010 Characterization of a rabies virus isolate from a ferret badger (*Melogale moschata*) with unique molecular differences in glycoprotein antigenic site III. Virus Res. 149:143–151 20109507 10.1016/j.virusres.2010.01.010

[5] Yu J Li H Tang Q Rayner S Han N Guo Z Liu H Adams J Fang W Tao X Wang S Liang G 2012 The spatial and temporal dynamics of rabies in China. PLoS Negl. Trop. Dis. 6:e1640. doi:10.1371/journal.pntd.0001640 10.1371/journal.pntd.0001640 PMC3341336 22563518

[6] Dietzschold B Wunner WH Wiktor TJ Lopes AD Lafon M Smith CL Koprowski H 1983 Characterization of an antigenic determinant of the glycoprotein that correlates with pathogenicity of rabies virus. Proc. Natl. Acad. Sci. U. S. A. 80:70–74 6185960 10.1073/pnas.80.1.70 PMC393311

[7] Seif I Coulon P Rollin PE Flamand A 1985 Rabies virulence: effect on pathogenicity and sequence characterization of rabies virus mutations affecting antigenic site III of the glycoprotein. J. Virol. 53:926–934 2579247 10.1128/jvi.53.3.926-934.1985 PMC254728

[8] Faber M Faber ML Papaneri A Bette M Weihe E Dietzschold B Schnell MJ 2005 A single amino acid change in rabies virus glycoprotein increases virus spread and enhances virus pathogenicity. J. Virol. 79:14141–14148 16254349 10.1128/JVI.79.22.14141-14148.2005 PMC1280225

[9] Morimoto K McGettigan JP Foley HD Hooper DC Dietzschold B Schnell MJ 2001 Genetic engineering of live rabies vaccines. Vaccine 19:3543–3551 11348722 10.1016/s0264-410x(01)00064-0

[10] Takayama-Ito M Inoue K Shoji Y Inoue S Iijima T Sakai T Kurane I Morimoto K 2006 A highly attenuated rabies virus HEP-Flury strain reverts to virulent by single amino acid substitution to arginine at position 333 in glycoprotein. Virus Res. 119:208–215 16473429 10.1016/j.virusres.2006.01.014

[11] Faber M Faber ML Li J Preuss MA Schnell MJ Dietzschold B 2007 Dominance of a nonpathogenic glycoprotein gene over a pathogenic glycoprotein gene in rabies virus. J. Virol. 81:7041–7047 17459937 10.1128/JVI.00357-07 PMC1933278

[12] Tao L Ge J Wang X Zhai H Hua T Zhao B Kong D Yang C Chen H Bu Z 2010 Molecular basis of neurovirulence of Flury rabies virus vaccine strains: importance of the polymerase and the glycoprotein R333Q mutation. J. Virol. 84:8926–8936 20538851 10.1128/JVI.00787-10 PMC2919041

